# The Association between Mood, Inhibitory Control and Depressive Symptoms: An Ecological Momentary Assessment Study

**DOI:** 10.1192/j.eurpsy.2023.901

**Published:** 2023-07-19

**Authors:** M. Nahum, N. Yitzhak, O. Shimony-Mazar, N. Oved, O. Bonne

**Affiliations:** 1 Faculty of Medicine; 2 Department of Psychology, The Hebrew University of Jerusalem; 3 Department of Psychiatry, Hadassah Medical Center, Jerusalem, Israel

## Abstract

**Introduction:**

Cognitive models of depression highlight the role of inhibitory control - the cognitive control ability which supports our goal directed behavior – as key and even causal feature of the disorder. According to these models, deficits in inhibitory control prevent the exclusion of irrelevant negative information, leading to rumination and sustained negative mood which result in depressive episodes. However, the scientific evidence linking deficits in inhibitory control to depression is thus far mixed. Moreover, although one’s inhibitory control ability may fluctuate, it is often assessed using a single-time measurement in the lab.

**Objectives:**

Here we aimed to assess the association between intra-individual fluctuations in inhibitory control measured in ecological settings, daily mood states, and depressive symptoms.

**Methods:**

N=106 participants (Mean age: 38 ± 10 years; range: 19-62 years; 68% female) reported their depressive symptoms (using the PHQ-9 scale) and completed a mobile version of the Go-NoGo inhibition task at baseline. They then completed a 5-day ecological-momentary-assessment (EMA) protocol, in which they reported their current mood (using the IMS-12 scale) and performed a shortened version of the Go-NoGo task twice/day using a mobile application. Depressive symptoms were assessed again following the 5-day EMA. Hierarchical-linear-modeling (HLM) was applied to examine the association between momentary IC and mood, with post-EMA depressive symptoms as a moderator. Inhibitory control was included as a time-varying predictor for mood in the 1^st^ step, and depressive symptoms post-EMA and their interaction with inhibition were included in the 2^nd^ step.

**Results:**

At baseline, there were no correlations between depressive symptoms and inhibitory control (*r_p_* = .035, n.s). However, individuals with elevated depressive symptoms demonstrated worse and more variable inhibition performance over time (*r_p_* = .29, *p* = .002), as captured in the EMA measures. In addition, participants with more variable inhibitory control performance over time also reported more depressive symptoms at the end of the 5-day period (*r_p_* = .27, *p* = .006). Finally, post-EMA depressive symptoms moderated the association between momentary inhibitory control and daily mood, such that reduced inhibition was associated with more negative mood only for those with lower, but not with higher, depressive symptoms (Figure 1).

**Image:**

**Figure:**
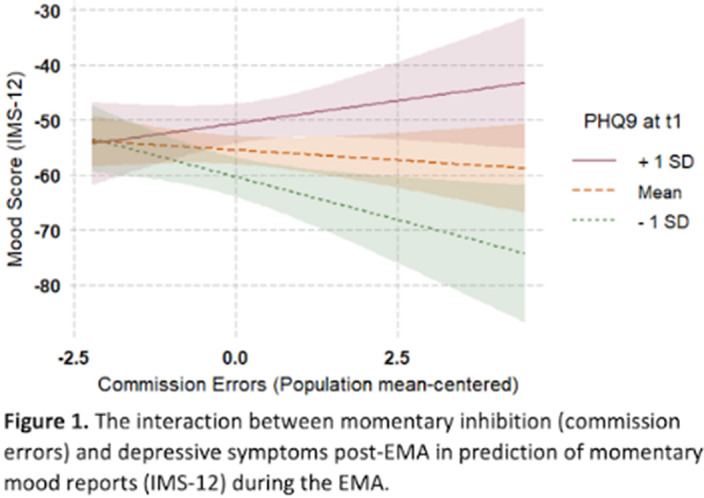

**Conclusions:**

Variable, rather than mere reduced inhibitory control is related to depressive symptoms. Moreover, the role of inhibition in modulating mood differs in non-depressed vs. depressed individuals. These findings contribute to our understanding of inhibition and mood in real life and help account for some of the discrepant findings related to cognitive control models of depression. Future investigations should examine the validity of these outcomes in other, clinical samples.

**Disclosure of Interest:**

None Declared

